# From scraped to published

**DOI:** 10.1016/j.patter.2024.100953

**Published:** 2024-03-08

**Authors:** Alejandra Alvarado, Andrew L. Hufton

**Affiliations:** Scientific Editor, *Patterns*; Editor-in-Chief, *Patterns*

## Main text

Large datasets collected from the internet are widely used in machine learning research and have been crucial in the development of many foundational AI models. Such datasets, however, pose special legal and ethical challenges. These challenges extend not just to researchers generating such datasets, but also to users who may have had no hand in the original collection process. At *Patterns*, we frequently encounter authors who have used web-scraped datasets but are unclear on how to meet the journal’s transparency and reproducibility requirements. These cases can raise complex questions regarding data origin, copyright, and ethics (see Paullada et al., 2021, *Patterns*) for what may otherwise appear to be straightforward research articles. Here, we offer our authors and readers some practical recommendations for publishing science based on web-scraped data. Our key message is that authors need to take responsibility for what they have used, even, and especially, when complex issues arise.

Our first and most important recommendation is to *inform yourself*. Learn as much as possible about each dataset with which you work. Content scraped from the web should be assumed to be protected by copyright, unless otherwise stated, and therefore researchers working with such content also need to understand copyright exemptions in their country and how those exemptions might apply to their research case. For a detailed discussion of how the “fair use” concept in US law may apply to foundational AI models, see Henderson et al. (2023 arXiv). Researchers also need to understand the limits of copyright exemptions in their jurisdiction. For example, the fact that “fair use” or similar exemptions may grant you the right to mine copyright material to build a model does not necessarily grant you the right to store and share that material.

Casting further uncertainty on this already complex topic are a number of lawsuits underway filed by plaintiffs alleging that a machine learning model trained on their content was infringing on their rights (see for example, Grynbaum and Mac, 2023, *New York Times*). There is a lack of laws and directives that deal specifically with web-scraped data and related technology, and legal scholars indicate that law and technology will have to evolve together for policies to develop (Henderson et al. 2023 arXiv). For now, researchers would be wise to be cautious.

In addition to issues of copyright, users of web-scraped content also need to consider ethical and privacy issues that arise when personal information may be included in the dataset. Datasets of images and text collected from the internet can violate personal privacy or propagate bias and damaging information (see, for example, Prabhu and Birhane, 2021, WACV). Facial image datasets in particular are under scrutiny for their potential abuse and lack of proper consent processes (Metz, 2019, *New York Times*).

Then, what can one do when using or creating a dataset with information scraped from the web? As mentioned above, inform yourself, and then be transparent about each step in your research process, from data creation and data analysis to data distribution and publication. Rely on your research institution and legal experts for guidance whenever complicated questions arise. When preparing a manuscript, keep these recommendations in mind:

(1) *Know your data.* Know the copyright exemption used to justify collection of the data, as well as what processes, if any, were used to obtain consent from data subjects or creators.
(2) *Be transparent.* Clearly describing the origin, limitations, and biases of a dataset will make your work all the more accessible to your peers and the community in general.
(3) *Be critical.* The fact that a certain dataset is commonly used by researchers does not mean that the dataset is free of issues or that its origin is indisputable.
(4) *Ask questions.* Every case is different. When details regarding dataset creation are not clear, we recommend contacting the individuals who originally collected the data from the web. When in doubt, we recommend asking your institution for ethical and legal advice.
(5) *Know when to say “no”.* This one goes with being critical; if validating the provenance or legality of a dataset reveals issues, it may be best to not use these data and find better alternatives. Avoid using and propagating datasets whose use is now deprecated by community norms (see Noorden, 2020, *Nature*).
(6) *Be proactive.* Finally, if concerns arise, we recommend taking responsible action early and involving ethical or legal experts as needed.

We hope that these tips will help our authors’ research go from scraped to published in a manner that is rigorous, transparent, and ethical. While laws, regulations, and research community expectations are still developing, individual changes in our attitudes toward data can have an immediate and far-reaching impact, helping ensure that we are publishing science based on ethically sourced data.

